# Determinants of developmental outcomes for children under 3 years in a rural setting, Kenya

**DOI:** 10.4102/jphia.v16i1.822

**Published:** 2025-05-21

**Authors:** Beatrice A. Oyugi, Silas O. Onyango, Henry Athiany, Gideon M. Kikuvi

**Affiliations:** 1 Department of Environmental Health and Disease Control, School of Public Health, Jomo Kenyatta University of Agriculture and Technology, Nairobi, Kenya; 2 Department of Human Development, Division of Early Childhood Development, Africa Population and Health Research Center, Nairobi, Kenya; 3 Department of Statistics and Actuarial Sciences, School of Mathematical and Physical Sciences, Jomo Kenyatta University of Agriculture and Technology, Nairobi, Kenya

**Keywords:** early childhood development, development outcomes, development domains, development delays, caregiving

## Abstract

**Background:**

Most children from low- and middle-income countries (LMICs) are at risk of poor development. Poor developmental outcomes are associated with later poor schooling and labour outcomes. Previous literature has documented a range of factors that influence children’s development. However, these factors are not well established in rural settings.

**Aim:**

The current study aims at assessing the determinants of developmental outcomes among children under 3 years.

**Setting:**

Siaya County, Kenya.

**Methods:**

A cross-sectional study of children under 3 years and their caregivers, attending Child Welfare Clinic (CWC) at tier 3 health facilities in a rural setting in Kenya, was conducted. A total of 149 children were randomly selected to participate in the study and had their development assessed using the third version of the Ages and Stages Questionnaire (ASQ-3). We assessed the association between ASQ-3 scores and environmental, cultural and individual-level factors in crude and adjusted linear regression models.

**Results:**

Being married and being employed showed the strongest positive associations with child development while earning less than $100.00 per month has a negative association with children’s development for individual-level factors. In addition, exposing children to opportunities for early learning had a greater effect on the child’s outcomes. Also, responsive caregiving showed higher scores for children’s developmental outcomes.

**Conclusion:**

The study suggests a positive association between child development outcomes and caregivers’ socio-demographic characteristics. There is also an association between responsive caregiver-child interactions and child development outcomes. Programmes that create an enabling environment for caregivers to provide a stimulating environment for their children may help children to thrive, improving their development outcomes.

**Contribution:**

This study contributes to the body of knowledge on the important roles of caregivers in enhancing their children’s optimal development.

## Introduction

The first 1000 days of life – the period from pregnancy to a child’s second birthday represents the most critical time for a child’s development, as the brains develop most rapidly during this period, laying the foundation for the achievement of critical developmental milestones necessary for future learning and development.^1,2^ Recent findings indicate that an estimated 250 million children under the age of five low- and middle-income countries (LMICs) are at risk of poor development,^3^ and up to 66% of these children live in rural or poor settings in sub-Saharan Africa (SSA).^4^ Poor child development outcome during this critical period is associated with later poor schooling and economic outcomes, perpetuating the intergenerational cycle of poverty.^5^

Previous studies have established that caregiver-child interactions are highly beneficial for early childhood development and have positive effects that last for a lifetime.^4^ Child stimulation, which involves spending quality time with the child in age-appropriate activities such as play, touching, talking, smiling and following the child’s lead in what interests them, is known to help build strong neural connections that support the growing brain of the child, laying a solid foundation for future learning.^6^ Various factors that range from caregiver and child characteristics such as age of caregiver-child, caregiver education level, marital status and income levels affect children’s development outcomes.^7,8^ Other factors that could affect developmental outcomes are exposure to early learning opportunities that allow the child to learn through play as they explore their environment and the quality of caregiver-child interactions.^9^ Understanding the determinants of developmental delays in children is important in designing interventions that improve such outcomes, enabling children to thrive and achieve their full developmental potential.^10^

There are limited studies that relate developmental outcomes and individual, cultural and environmental factors in rural settings in Kenya. The current study fills this gap by determining the association between key factors and how they determine the developmental outcomes of children. The result from this study is intended to contribute to the body of knowledge on determinants of developmental outcomes among children, and programmers would use this to design interventions that improve child developmental outcomes.

## Research methods and design

### Research design

A cross-sectional study design was used for this study. This was deemed appropriate as the intent of the study was to collect data on the determinants of developmental outcomes without influencing them. The choice of the design was also necessitated by logistical issues such as budget limitations. The study employed quantitative data collection techniques as the main approach. This design was suitable for testing the association between developmental outcomes among children with other variables such as individual-level or caregiver and child-level factors, environmental or child stimulation factors and cultural factors or caregiving practices among children aged below 3 years in a rural setting in Kenya.

### Study setting

The study was carried out in Siaya County, situated in the western part of Kenya, where fishing and small-scale farming are the main economic activities. As of 2019, the County had a population of 993 183 people with a 1.7% growth rate.^11^ Women of reproductive age make up 23% of the population, and the fertility rate is 4.2, slightly higher than the national rate of 3.9. Children under 5 years account for 17% of the population, with 3% being under 1 year.^12^ Approximately 24.7% of children under 5 years are stunted, while 8% are underweight.^13^

### Sample size determinations and sampling procedure

#### Sample size determination

The study determined the sample size using Cochran’s method with a 95% confidence level and a 5% sampling error. The formula we used (Equation 1):


$$n=z^{2}\left[P(1-P)/d^{2}\right]$$ [Eqn 1]

where *z* is 1.96, *P* is 0.1 based on previous studies^14^ and *d* is 0.05. The calculated sample size was 138 but considering a 10% non-response rate, 152 respondents were interviewed.

#### Sampling procedure

In stage one, tier 3 health facilities were purposively selected for their adequate distribution; client volumes and maternal, neonatal and child health services provision. Four facilities were randomly chosen from a pool of 22. Children aged below 3 years were the main participants in this study. The number of study participants selected from each facility was weighted to the number of children under 3 years accessing Child Welfare Clinic (CWC) services in the previous year. In stage two, study participants were selected through systematic random sampling from Maternal, Newborn and Child Health (MNCH) clinics in the designated sites, using CWC registers as sampling frames. Data collection lasted for 3 weeks.

### Recruitment and training of data collectors

Data were collected by the first author with the help of research assistants who had a college level of education in social sciences and who had experience in conducting field interviews. Interviewers were carefully trained in the interview tools and the use of an Open Data Kit (ODK) for data collection. All interviewers were fluent in English and Kiswahili as well as Dholuo, which is the local language of the study area.

Research assistants were stationed at the MNCH clinics of the study sites. All study participants were interviewed on-site. The research assistants administered consent forms to potential study participants. Those who met the inclusion criteria and consented to take part in the study were enrolled and interviewed. A total of 149 caregivers of children aged below 3 years consented to participate in the study and were interviewed.

### Exposure and outcome variables

The main exposure variable for this study was developmental outcomes assessed using the third edition of the Ages and Stages Questionnaire (ASQ)-3.^15^ The ASQ-3 is an easy-to-use, reliable and valid screening instrument used to identify potential developmental delays among children of 2 months to 5 years who need further assessment.^16^ It is a parental-reported tool that assesses children’s performance along five developmental domains: fine motor skills, gross motor skills, language or communication, cognitive or problem-solving and personal social domains of development.

The ASQ-3 has been validated and is used to assess child development in a similar study setting.^17,18^ In this study, the tool was administered through caregivers’ self-reported questions. Six questions were asked under each domain, and responses were recorded as ‘yes’ = 10 points if the child was able to perform an activity, sometimes if the child tried and failed but the caregiver reported that the child could perform the activity, and ‘no’ = 0 points if the child was unable to perform the activity. Scores corresponding to the responses to each item in each domain were summed to obtain an aggregate score (ranging from 0 to 60). Scores were reported across five domains of development: fine motor skills, gross motor skills, language or communication, cognitive or problem-solving and personal social domains. The overall score for each child indicating developmental outcomes was obtained by summing the domain totals and dividing by 5 (total number of domains) to give a mean score. The ASQ-3 provides a summary determined by both scores and individual responses on each development domain.

The determinants of developmental delays were categorised into three: individual-level factors or socio-demographic determinants, information was obtained about the age and sex of the child and caregiver socio-economic factors. Caregiver education was conceptualised as primary (those who reported education up to 8 years of primary education) and secondary and above for those who reported some secondary and post-secondary education. Caregiver marital status was constructed as married or not (for those who were single, separated or divorced). Caregiver income was categorised as no income, income below $100.00 and income above $100.00 per month. Caregiver occupation was categorised as a caregiver not employed or employed (for those who reported any formal or informal employment). Environmental factors or child stimulation was categorised as exposure to early learning opportunities. Information was obtained about whether the caregiver engaged the child in play, naming objects, colours and shapes of objects in their environment. Caregivers were also assessed on whether they taught their children counting and sounds.

Cultural factors or responsive caregiving was categorised as caregiving practices. Information was obtained about whether the caregiver had been pleased with the child in the previous week, the involvement of the child’s father or other male caregivers in caring for the child, how the caregiver responds when the child does something that the caregiver does not want the child to do, what the caregiver does during child’s feeding time and whether the caregiver slapped or spanked the child. Child’s health (caregiver knowledge of whether the child is growing well, how the caregiver makes the child eat when the child does not want to) and child safety (who takes care of the child when the caregiver must leave or is away).

### Statistical analysis

Descriptive analysis was conducted where frequencies and percentages were reported for categorical variables while mean and standard deviations (s.d.) were reported for continuous variables. Unadjusted linear regression was carried out to determine the association between individual factors, cultural factors and environmental factors associated with child developmental outcomes separately. For individual-level factors, we fit a model for each of the factors separately and another model for all the factors together. A multivariate linear regression was also carried out to determine the association between cultural and environmental factors and their association with children’s development by including all the covariates. For all the models, a 95% confidence interval (CI) and *p*-values were reported. A *p*-value of < 0.05 was considered to be statistically significant.

### Ethical considerations

Ethical clearance to conduct this study was obtained from the University of Eastern Africa, Baraton Research Ethics Committee (No. UEAB/14/5.2018). Informed consent was obtained from participants, who were informed about the purpose, process, benefits and risks of the study. Consent forms were signed by willing participants, but refusal was allowed without explanation or penalty. Privacy and confidentiality were respected by trained field interviewers. Participation in the study was voluntary, with no harm inflicted on caregivers or children.

## Results

A total of 149 caregiver-baby pairs were contacted, and all of them consented to participate in the study. Results from 135 respondents were completed and analysed. Table 1 presents the socio-demographic characteristics of the study population. The children enrolled in the study were almost evenly distributed in terms of sex, with 71 (52.6) being males. The mean age of children was 12 ± 9.5 months while the mean age of caregivers was 26.5 ± 7.5 years. Almost 60% of the caregivers interviewed had some primary education and 74.8% were married. In addition, nearly a third of the caregivers (34.8%) had no monthly income, while 54.1% had income less than $100.00 per month.

**TABLE 1:** Descriptive statistics (socio-demographics) (*N* = 135).

Child/caregiver’s characteristics	*n*	%	Mean	s.d.
Child female	64	47.4	-	-
Child age (months)	-	-	12.1	9.5
Caregiver age (years)	-	-	26.5	7.5
**Caregiver education**				
Primary	80	59.3	-	-
Secondary and above	55	40.7	-	-
Caregiver employed	95	70.4	-	-
Caregiver married	101	74.8	-	-
**Caregiver monthly income**				
No income	47	34.8	-	-
Below $100.00	73	54.1	-	-
Above $100.00	15	11.1	-	-

s.d., standard deviation.

As presented in Table 2, the mean ASQ scores for children was 48.9. On average, children had lower scores in personal social (44.8) and higher scores in communication domains (51.1). Less than half (46.8%) of the children had been exposed to stimulation activities, while less than a quarter (21.5%) were exposed to responsive care activities.

**TABLE 2:** Descriptive statistics (outcome and exposure variables) (*N* = 135).

Variables	Mean	s.d.	*n*	%
Outcome variables				
Ages and stages	48.9	9.1	-	-
Communication domain	51.1	11.4	-	-
Gross motor domain	50.5	14.5	-	-
Fine motor domain	48.6	12.0	-	-
Problem-solving domain	49.4	14.0	-	-
Personal social domain	44.8	12.8	-	-
**Exposure variables**				
Environmental factors/child stimulation				
No	-	-	72	53.3
Yes	-	-	63	46.8
Cultural factors/responsive caregiving				
No	-	-	106	78.5
Yes	-	-	29	21.5

s.d., standard deviation.

The association between cumulative child development delays and socio-demographic/individual-level factors is presented in Table 3. Being married and being employed showed the strongest positive associations with child development. The results show that caregivers who were married had a 0.50 s.d., higher ASQ scores (95% CI: 0.09–0.90) after adjusting for possible covariates. Also, caregivers who were employed had a 0.61 s.d. (95% CI: 0.09–1.12) higher score compared to those who were not employed. There is, however, a negative association between income of less than $100.00 and developmental outcomes of children at –0.61 s.d. (95% CI: –1.09 to –0.12). The results imply that being married and having a form of employment, whether formal or informal, has a positive contribution to the development of children.

**TABLE 3:** Linear regression for the associations between overall child development (ASQ) and individual factors.

Individual factors	Unadjusted		Adjusted	
	s.d.	95% CI	s.d.	95% CI
Caregiver age	0.03***	0.01 to 0.06	0.02	-0.01 to 0.04
Caregiver education	0.31*	-0.03 to 0.66	0.23	-0.11 to 0.57
Caregiver married	0.65***	0.27 to 1.03	0.50**	0.09 to 0.90
Income below $100	-0.04	-0.41 to 0.32	-0.61**	-1.09 to -0.12
Income above $100	0.67**	0.09 to 1.24	-0.38	-1.07 to 0.32
Caregiver employed	0.48***	0.12 to 0.85	0.61**	0.09 to 1.12
Child age	0.02**	0.01 to 0.04	0.02*	-0.00 to 0.04
Child female	-0.06	-0.41 to 0.28	-0.01	-0.33 to 0.31
Observations	135.00	-	135.00	-

s.d., standard deviation; CI, confidence interval.

* , significance at *p* < 0.1;

** , significance at *p* < 0.05;

*** , significance at *p* < 0.01.

Results on crude and adjusted association between developmental delays and environmental factors are presented in Table 4. In the unadjusted model, child stimulation was associated with 0.97 s.d. (95% CI: 067–1.27), higher ASQ-3 mean scores. After adjusting for covariates, children who were stimulated had 0.85 s.d. (95% CI: 0.67–1.27), higher mean scores. The results imply that children who are exposed to early learning opportunities through routine caregiver-child interactions are more likely to develop optimally.

**TABLE 4:** Unadjusted and adjusted linear regression for the associations between overall child development (ASQ) and environmental factors.

Variables	Unadjusted		Adjusted	
	s.d.	95% CI	s.d.	95% CI
Child stimulation	0.97 ***	0.67 to 1.27	0.85 ***	0.48 to 1.22
Caregiver age	-	-	0.02	-0.00 to 0.04
Caregiver education	-	-	0.17	-0.15 to 0.49
Caregiver married	-	-	0.42 **	0.05 to 0.80
Income below $100	-	-	-0.33	-0.79 to 0.14
Income above $100	-	-	-0.24	-0.89 to 0.41
Caregiver employed	-	-	0.33	-0.17 to 0.83
Child age	-	-	-0.01	-0.03 to 0.01
Child female	-	-	-0.04	-0.34 to 0.25
Constant	-0.45 ***	−0.66 to -0.25	-1.73 ***	-2.74 to -0.72
Observations	135.00	-	135.00	-

Note: In the adjusted analysis, we controlled for socio-demographic characteristics.

s.d., standard deviation; CI, confidence interval.

*** , *p* < 0.01;

** , *p* < 0.05;

*, *p* < 0.1 at 95% confidence levels.

Results for unadjusted and adjusted association between cultural factors or responsive caregiving and developmental delays are presented in Table 5. Results show that children who received responsive care had a 0.45 higher ASQ-3 mean score (95% CI: 0.04–0.86) in the unadjusted model. In the adjusted model, results show that responsive caregiving was associated with a 0.56 higher ASQ-3 mean score (95% CI: 0.16–0.95). The results imply that when caregivers are sensitive and responsive to the needs of their children and are actively involved in their well-being, then children are more likely to achieve better development outcomes.

**TABLE 5:** Unadjusted and adjusted linear regression for the associations between overall child development (ASQ) and cultural factors (responsive caregiving).

Variables	Unadjusted		Adjusted	
	Mean	95% CI	Mean	95% CI
Responsive caregiving	0.45**	0.04 to 0.86	0.56***	0.16 to 0.95
Caregiver age	-	-	0.02*	-0.00 to 0.04
Caregiver education	-	-	0.18	-0.16 to 0.51
Caregiver married	-	-	0.43**	0.04 to 0.83
Income below $100	-	-	-0.65***	-1.12 to -0.17
Income above $100	-	-	-0.44	-1.12 to 0.24
Caregiver employed	-	-	0.61**	0.10 to 1.11
Child age	-	-	0.02**	0.00 to 0.04
Child female	-	-	-0.08	-0.39 to 0.23
Constant	-0.10	−0.29 to 0.09	-1.86***	-2.91 to -0.81
Observations	135.00	-	135.00	-

Note: In the adjusted analysis, we controlled for socio-demographic characteristics.

CI, confidence interval.

*** , *p* < 0.01;

** , *p* < 0.05;

* , *p* < 0.1 at 95% confidence levels.

## Discussion

The determinants of developmental outcomes among children aged below 3 years were assessed. Results show that socio-demographic factors of the caregiver, particularly their marital status, are associated with child development outcomes. Caregivers who are married and raise their children together are likely to have children who develop optimally compared to those who are not married and raise their children as single parents. These findings are in line with the available literature,^8,19^ which has shown that children who grow up under the care of single parents have poorer developmental outcomes.

The study also established an association between caregiver income levels and child developmental outcomes. Children whose caregivers have no regular source of income have poor developmental outcomes. These findings confirm what is documented in the literature^4,20^ that low income is associated with extreme poverty, which increases exposure to other adversities such as food insecurity, toxic stress, child abuse and neglect, and these compromise the development of the child, resulting in poor outcomes.

Consistent with existing literature,^21^ the study results suggest that exposing children to age-appropriate stimulation activities that allow them to explore their environments and interact with their caregivers, objects and playthings of different textures, colours and shapes may improve child outcomes in all domains of development, including fine motor, gross motor, personal social, problem solving and communication. When caregivers deliberately engage their children in age-appropriate early learning opportunities and encourage them to play as a way of exploring their environment and learning new things, children tend to have better developmental outcomes, which prepare them for early-grade success and set them on a positive trajectory for future learning and development.

The study also suggests that warm caregiver-child interactions characterised by caregiver sensitivity and responsiveness to the needs of the child are associated with positive developmental outcomes in their personal, social and fine motor domains of development. The results suggest that ensuring a warm stimulating caregiving environment would enhance children’s social-emotional development, equipping them with self-regulation skills necessary for success in life. These findings are in line with previous research that has shown that children acquire social-emotional skills when they experience warm and responsive care.^17^ In line with existing literature, this study also established that ASQ-3 is an easy-to-use screening tool that can be used in African settings.^22^ The main limitation of this study is the reliance on primary caregivers’ self-report, and this may have social desirability bias.

## Conclusion

The study suggests a positive association between child developmental outcomes and caregivers’ socio-demographic characteristics. There is also an association between caregiver-child interactions and child developmental outcomes. Programmes that improve caregivers’ socio-economic status and create an enabling environment for the provision of age-appropriate child stimulation and responsive care may help children to thrive, improving their developmental outcomes.

## Appendix 1

**TABLE 1-A1:** Linear regressions for the associations between domain-specific development (ASQ) and individual-level factors.

Variables	Communication		Gross motor		Fine motor		Problem-solving		Personal social	
	s.d.	95% CI	s.d.	95% CI	s.d.	95% CI	s.d.	95% CI	s.d.	95% CI
Caregiver age	0.02*	−0.00 to 0.05	0.00	−0.02 to 0.03	0.01	−0.01 to 0.03	0.03**	0.01 to 0.06	0.00	−0.03 to 0.03
Caregiver education	0.10	−0.26 to 0.46	0.09	−0.28 to 0.46	0.21	−0.15 to 0.57	0.35*	−0.00 to 0.70	0.05	−0.32 to 0.41
Caregiver married	0.44**	0.01 to 0.86	0.34	−0.09 to 0.78	0.43**	0.01 to 0.85	0.26	−0.15 to 0.68	0.30	−0.13 to 0.73
Income below $100	−0.49*	−1.00 to 0.02	−0.38	−0.91 to 0.14	−0.47*	−0.98 to 0.03	−0.54**	−1.04 to -0.04	−0.25	−0.77 to 0.26
Income above $100	−0.22	−0.94 to 0.51	−0.10	−0.84 to 0.65	−0.61*	−1.33 to 0.12	−0.49	−1.21 to 0.23	0.06	−0.68 to 0.80
Caregiver employed	0.32	−0.22 to 0.86	0.37	−0.19 to 0.92	0.66**	0.12 to 1.20	0.56**	0.02 to 1.09	0.23	−0.32 to 0.78
Child age	0.01	−0.01 to 0.03	0.01	−0.00 to 0.03	0.01	−0.01 to 0.03	−0.00	−0.02 to 0.02	0.02**	0.00 to 0.04
Child female	−0.09	−0.42 to 0.24	−0.07	−0.41 to 0.27	0.11	−0.22 to 0.44	0.11	−0.21 to 0.44	−0.10	−0.44 to 0.24
Constant	−1.41**	−2.54 to -0.28	−0.90	−2.06 to 0.26	−1.78***	−2.91 to -0.66	−1.94***	−3.06 to -0.82	−0.76	−1.91 to 0.39
Observations	135.00	-	135.00	-	135.00	-	135.00	-	135.00	-

s.d., standard deviation; CI, confidence interval.

## Appendix 2

**TABLE 1-A2:** Adjusted associations between domain-specific development (ASQ) and environmental factors.

Variables	Communication		Gross motor		Fine motor		Problem-solving		Personal social	
	s.d.	95% CI	s.d.	95% CI	s.d.	95% CI	s.d.	95% CI	s.d.	95% CI
Child stimulation	0.85***	0.46 to 1.23	0.59***	0.17 to 1.01	0.35*	−0.06 to 0.76	0.54***	0.14 to 0.95	0.68***	0.28 to 1.09
Caregiver age	0.02*	−0.00 to 0.04	0.00	−0.02 to 0.03	0.01	−0.01 to 0.03	0.03**	0.01 to 0.05	−0.00	−0.02 to 0.02
Caregiver education	0.04	−0.29 to 0.38	0.05	−0.31 to 0.41	0.19	−0.17 to 0.54	0.31*	−0.04 to 0.66	−0.00	−0.35 to 0.35
Caregiver married	0.37*	−0.03 to 0.76	0.29	−0.13 to 0.72	0.40*	−0.02 to 0.82	0.22	−0.19 to 0.63	0.24	−0.17 to 0.66
Income below $100	−0.21	−0.71 to 0.28	−0.19	−0.72 to 0.34	−0.36	−0.88 to 0.16	−0.36	−0.87 to 0.15	−0.03	−0.54 to 0.49
Income above $100	−0.08	−0.76 to 0.60	−0.00	−0.73 to 0.73	−0.55	−1.28 to 0.17	−0.40	−1.11 to 0.30	0.17	−0.54 to 0.89
Caregiver employed	0.05	−0.48 to 0.57	0.18	−0.38 to 0.74	0.55*	−0.01 to 1.10	0.38	−0.16 to 0.92	0.01	−0.53 to 0.56
Child age	−0.01	−0.03 to 0.01	−0.00	−0.02 to 0.02	0.00	−0.02 to 0.03	−0.02*	−0.04 to 0.00	0.01	−0.02 to 0.03
Child female	−0.13	−0.44 to 0.18	−0.09	−0.42 to 0.24	0.09	−0.23 to 0.42	0.09	−0.23 to 0.41	−0.13	−0.45 to 0.20
Constant	−1.22**	−2.28 to -0.16	−0.77	−1.91 to 0.36	−1.71***	−2.83 to -0.58	−1.82***	−2.92 to -0.73	−0.61	−1.72 to 0.50
Observations	135.00	-	135.00	-	135.00	-	135.00	-	135.00	-

s.d., standard deviation; CI, confidence interval.

## Appendix 3

**TABLE 1-A3:** Adjusted associations between domain-specific development (ASQ) and cultural factors.

Variables	Communication		Gross motor		Fine motor		Problem-solving		Personal social	
	s.d.	95% CI	s.d.	95% CI	s.d.	95% CI	s.d.	95% CI	s.d.	95% CI
Responsive caregiving	0.20	−0.23 to 0.62	0.34	−0.10 to 0.77	0.56***	0.15 to 0.97	0.30	−0.12 to 0.71	0.58***	0.16 to 1.00
Caregiver age	0.02*	−0.00 to 0.05	0.00	−0.02 to 0.03	0.01	−0.01 to 0.04	0.03**	0.01 to 0.06	0.00	−0.02 to 0.03
Caregiver education	0.08	−0.28 to 0.44	0.06	−0.31 to 0.43	0.16	−0.19 to 0.51	0.32*	−0.04 to 0.67	−0.01	−0.37 to 0.35
Caregiver married	0.41*	−0.01 to 0.84	0.30	−0.13 to 0.74	0.37*	−0.05 to 0.78	0.23	−0.19 to 0.65	0.23	−0.19 to 0.65
Income below $100	−0.51*	−1.02 to 0.00	−0.41	−0.93 to 0.11	−0.52**	−1.01 to -0.02	−0.56**	−1.06 to -0.06	−0.30	−0.80 to 0.21
Income above $100	−0.24	−0.97 to 0.49	−0.14	−0.88 to 0.61	−0.67*	−1.38 to 0.04	−0.52	−1.24 to 0.19	−0.00	−0.72 to 0.72
Caregiver employed	0.32	−0.22 to 0.86	0.37	−0.19 to 0.92	0.66**	0.13 to 1.19	0.56**	0.02 to 1.09	0.23	−0.30 to 0.77
Child age	0.01	−0.01 to 0.03	0.02*	−0.00 to 0.04	0.02**	0.00 to 0.04	−0.00	−0.02 to 0.02	0.03***	0.01 to 0.05
Child female	−0.12	−0.45 to 0.22	−0.11	−0.45 to 0.23	0.04	−0.28 to 0.37	0.08	−0.25 to 0.41	−0.17	−0.50 to 0.16
Constant	−1.39**	−2.52 to -0.26	−0.87	−2.02 to 0.29	−1.73***	−2.83 to -0.62	−1.91***	−3.02 to -0.79	−0.70	−1.83 to 0.42
Observations	135.00	-	135.00	-	135.00	-	135.00	-	135.00	-

s.d., standard deviation; CI, confidence interval.
